# Neurodevelopmental Consequences of Pediatric Cancer and Its Treatment: The Role of Sleep

**DOI:** 10.3390/brainsci10070411

**Published:** 2020-07-01

**Authors:** Maria Paola Mogavero, Oliviero Bruni, Lourdes M. DelRosso, Raffaele Ferri

**Affiliations:** 1 Istituti Clinici Scientifici Maugeri, IRCCS, Scientific Institute of Pavia, 27100 Pavia, Italy; paola_mogavero@libero.it; 2 Department of Developmental and Social Psychology, Sapienza University, 00185 Rome, Italy; oliviero.bruni@uniroma1.it; 3 Pulmonary and Sleep Medicine, University of Washington, Seattle Children’s Hospital, Seattle, WA 98105, USA; lourdes.delrosso@seattlechildrens.org; 4 Sleep Research Centre, Department of Neurology I.C., Oasi Research Institute—IRCCS, 94018 Troina, Italy

**Keywords:** neurodevelopmental disorders, intellectual disability, neurocognitive deficit, sleep, cancer, tumor

## Abstract

Cognitive impairment is frequent in pediatric cancer, and behavioral and psychological disturbances often also affect children who have survived cancer problems. Furthermore, pediatric tumors are also often associated with sleep disorders. The interrelationship between sleep disorders, neurodevelopmental disorders and pediatric cancer, however, is still largely unexplored. In this narrative review we approach this important aspect by first considering studies on pediatric cancer as a possible cause of neurodevelopmental disorders and then describing pediatric cancer occurring as a comorbid condition in children with neurodevelopmental disorders. Finally, we discuss the role of sleep disorders in children with cancer and neurodevelopmental disorders. Even if the specific literature approaching directly the topic of the role of sleep in the complex relationship between pediatric cancer and neurodevelopmental disorders was found to be scarce, the available evidence supports the idea that in-depth knowledge and correct management of sleep disorders can definitely improve the health and quality of life of children with cancer and of their families.

## 1. Introduction

Cancer is the leading cause of disease-related death among children, although recent therapeutic advances have improved life expectancy; in fact, nearly 90% of children diagnosed with cancer survive at least five years after diagnosis and over 70% at ten years [1].

The most common tumors diagnosed in childhood are leukemias (acute lymphoblastic leukemia in 78% of cases), brain neoplasms (especially astrocytoma and non-Hodgkin’s lymphoma) and of the central nervous system (CNS), which represent 34%, 23% and 12% of all childhood cancers, respectively. Solid tumors that are not localized in the CNS are very rare. Age strongly influences cancer occurring in childhood: the highest incidence is in the age group under five years (characterized by rapid brain and functional development and, therefore, a period in which the brain is particularly vulnerable), and a second peak of incidence occurs during puberty [2].

In recent years, a close relationship between pediatric cancer and cognitive impairment has been established, as well as behavioral and psychological disturbances in children who have survived cancer problems [3,4,5,6,7]; it has been estimated that 40 to 100% of brain tumor survivors have neurocognitive problems [8]. Cancer treatment can also impact negatively on the child’s health, even after recovery [3,7,9,10].

Furthermore, several studies have been conducted concerning the association between pediatric tumors and sleep disorders, highlighting how these are frequent in children with malignancies [2,11,12], but there is a scarcity of data on the neurodevelopmental effects associated with both cancer and sleep in children. The aim of this narrative is therefore to shed light on the correlation between sleep disorders, neurodevelopmental disorders and pediatric neoplasms in children.

Data for this review consisted of empirical articles published in peer-reviewed journals between 2010 and February 2020. An extensive computer-assisted literature search was conducted using PubMed, selecting only studies involving humans. Several searches were carried out using the following terms in various combinations: “pediatric cancer”, “neurodevelopment”, “pediatric tumor”, “neurodevelopmental disorder”, “sleep”, “leukemia”, “pediatric leukemia”, and “adolescent cancer”. The search for the association between specific sleep disorders (insomnia, parasomnia, hypersomnia, narcolepsy, sleepwalking, night terrors, restless legs syndrome, periodic limb movement, somnambulism) and “pediatric cancer”, “tumor”, and “neurodevelopment” did not retrieve any records.

Excluded from consideration were book chapters, monographs, commentaries, review articles, dissertations, abstracts, letters to the editor, and any non-data-analytic or non-peer-reviewed reports. Duplicates were also excluded, and the reference list of the retrieved articles was reviewed and pertinent articles published during the same time period specified above were added to the final list of papers. However, only four articles [13,14,15,16] dealt with cancer, neurodevelopmental disorders and sleep at the same time; for this reason, the following sections of this paper discuss the topic by using all pertinent articles that approached various aspects of the problem, even if not focusing on the very specific topic of our search.

### 1.1. Pediatric Cancer as a Cause of Neurodevelopmental Disorders

The influence of pediatric tumors and their treatment on neural development and on behavioral and emotional outcomes, even in the long term, has only recently been investigated [3,4,5,6,7]. It has been reported that cancer survivors treated with radio-chemotherapy show a high rate of cognitive dysfunction, with attention deficits in 67% and memory deficits in 3–28% of cases, as well as disorders of executive functions and speed of elaboration [17,18].

The onset of psychological problems (such as post-traumatic stress disorder) is very frequent, which can negatively impact on school performance and daily performance, as well as determine a reduction in therapeutic adherence, with a consequent increase in morbidity and mortality [19].

The onset of post-traumatic stress disorder may be due both to the diagnosis of malignant disease (resulting in a psychological impact on the child and the family) and to the anti-cancer treatment [20]. The management of the psychological sphere in oncology, therefore, plays a predominant role and, considering the close correlation between sleep disorders and psychiatric and neurodevelopment problems [21], treating sleep disorders can also be very useful in the treatment of cancer, being able to positively influence the psychological and cognitive components. In recent years, it has in fact been shown that sleep deprivation can affect neurodevelopment in children and adolescents, with repercussions on physical and mental health also causing structural alterations of the brain circuits in the frontal and limbic region, involved in the circuits of emotion [21,22].

The neurobiological changes observed are largely attributed to the neurotoxic effects of anticancer treatments [7], with dose-dependent effects of these therapies on brain structure and function [23,24,25]; these harmful effects on neurodevelopment also occur in the case of therapies for the treatment of leukemia [3], solid CNS tumors [10], and solid tumors not localized in the CNS [9,26].

In the case of brain tumors, in addition to radio-chemotherapy treatment, the localization of the tumor can also affect the neurocognitive outcome, with infratentorial tumors associated with worse outcomes compared to those with a higher localization: a study compared verbal and non-verbal intellectual functions, working, visual and verbal memory, visual–spatial integration, attention, and social and emotional functions in two groups of children with brain tumors (subjected to resection of the tumor and at the same doses of radiotherapy), and taking into account the localization of the brain tumor. Children with infratentorial tumors had lower school scores (and a greater frequency of hearing deficits) than children with supratentorial cancer [27].

Cognitive and psychological problems related to the onset of cancer can also be influenced by the age of the child at the time of diagnosis [28] and by the timing with which radio-chemotherapy treatment is carried out, which is more harmful in the case of early treatment [10] and capable of damaging several subcortical regions involved in the integration of affective and motivationally relevant signals, such as the amygdala, the thalamus, the ventral striatum, the substantia nigra/ventral tegmental area and, finally, the hippocampus, which plays a key role in the circuits of memory and emotions connected to the aforementioned structures [29].

The brain in the first years of life is particularly vulnerable to the negative effects of treatment due to rapid cell proliferation, dendritic and axonal growth, and myelination, which occur in childhood and adolescence [10]. The maturation of the gray and white matter is damaged, with a consequent slower cognitive processing speed, as the glial progenitor cells (responsible for the formation of oligodendrocytes and astrocytes) and hippocampal cells (involved in the processes of neurogenesis) are particularly vulnerable to the effects of chemo and radiotherapy [30], both in patients with leukemia [3], and in patients with solid CNS tumors [10].

The damage is due to both direct cell toxicity, induced by chemotherapy, and to inflammation and oxidative stress (i.e., indirect toxicity), which seem to have a negative impact especially on the hippocampus and prefrontal regions, causing behavioral disorders, such as lack of self-control before and during adolescence [31,32].

As mentioned above, acute lymphoblastic leukemia (ALL), the form of neoplasm that occurs most frequently in children, is a cause of impaired neurodevelopment of the child [3,33], but only in recent years have the specific biological mechanisms acting on long-term neuronal integrity, induced by ALL and the therapies administered for this pathology, been specified.

A recent study evaluated patients aged between 8 and 21 years treated with a single chemotherapy protocol with methotrexate and demonstrated a brain connectome dysfunction; the results were consistent with a delay in neurodevelopment (especially in younger children), which could be associated with reduced recovery capacity, adaptability and flexibility of the brain network [34].

Functional magnetic resonance imaging studies conducted in children with ALL have also shown worse neurocognitive dysfunction in the case of early treatment and with a higher dosage of methotrexate, highlighting a reduction in the activity of the right temporal lobe and of the frontal and parietal lobes, bilaterally [35].

However, in children with ALL, the white matter seems to be damaged even before chemotherapy treatment [13]. A very interesting study also evaluated cerebrospinal fluid biomarkers, demonstrating a cytokine-mediated inflammatory mechanism that, once it passes the blood–brain barrier, may trigger a cascade of neurotoxic events.

An increase in tau protein was observed (suggestive of axonal damage) in association with a worsening of attention deficit and a reduction in intelligence quotient; an increase in glial fibrillary acidic protein (GFAP) concentration in patients with an altered allele of the apolipoprotein E (APOE) gene (associated with deficiency of attention); and an increase in leukoencephalopathy, with compromised white matter especially in the frontal (particularly in the dopaminergic circuits) and parietal [36] lobes. This study also showed an impairment of brain structures both before and after chemotherapy: glial damage was present at the diagnosis; after intrathecal chemotherapy, neuronal damage was triggered, which was worse in cases of higher methotrexate dosage, especially in patients with the Val allele in the catechol-O-methyltransferase (COMT) gene and early treatment [36].

Table 1 summarizes the main biological mechanisms of CNS damage occurring in the course of pediatric cancer.

**Figure 1:**
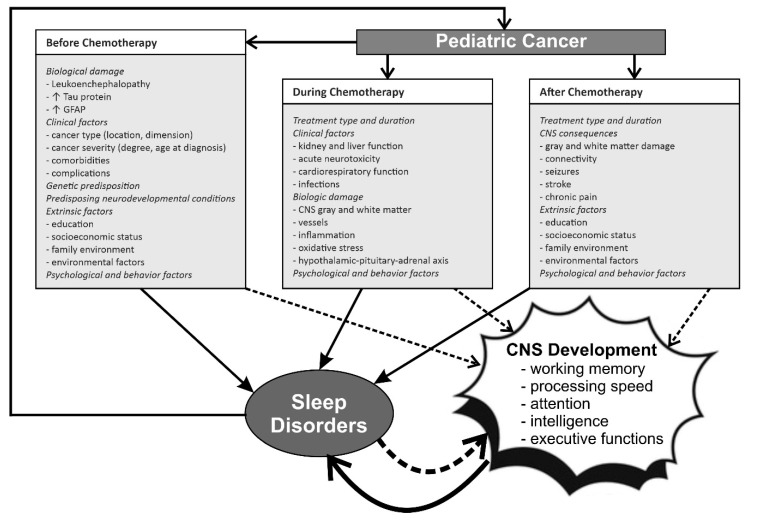
The complex relationship between pediatric cancer, chemotherapy, CNS development and sleep before, during, and after treatment. GFAP, glial fibrillary acidic protein. ↓ decrease; ↑ increase.

The relationship and interactions between pediatric cancer, chemotherapy, sleep and CNS development/damage and cognitive function are complex and schematically represented in Figure 1.

The above results highlight the need for interventions that prevent or manage cognitive impairment in pediatric ALL; in recent years, the results of cognitive behavioral therapy (CBT) and physical activity in pediatric patients with ALL or solid CNS tumors have been examined, with improvement of brain function and an increase in white matter and hippocampal volume [38,39,40]. These data underline that the treatment of psychological problems in children with cancer is also of fundamental importance because it involves a potential improvement in cognitive performance; on the other hand, it has been shown that correct sleep hygiene is very important for the protection of the physical and mental health of children and that health workers take care of this aspect in clinical practice [21].

The studies conducted on the effects of pediatric tumors not located in the CNS and their treatment on neuronal development are indeed very few (given the rarity of these tumors). However, even in this case, there seems to be evidence of damage related to neuro-inflammation and chemotherapy-induced damage of the blood–brain barrier [9,26]. Finally, in these types of tumors chemotherapy can damage the hypothalamic–pituitary–adrenal axis [37], altering hormonal structure and inducing growth problems and depressive symptoms [9].

Therefore, before or during radio-chemotherapy, an early treatment of psychological and cognitive problems seems to be appropriate in order to positively influence the maturation processes of the hippocampus and related structures and the developing neuronal plasticity [7]. It seems that the hippocampus is one of the few brain areas that shows active postnatal neurogenesis, which makes it particularly susceptible to changes induced by pediatric tumors [41].

### 1.2. Pediatric Tumors Arising in Comorbidity with Neurodevelopmental Disorders

Regarding the studies conducted on congenital neurological development anomalies in pediatric cancer and young adults, there are a few reports in the literature showing that 8–30% of pediatric oncology patients have mutations in cancer-predisposing genes [42].

There is a single study that expands previous reports on the associations of congenital anomalies with pediatric tumors by integrating neurocognitive deficits; this study examined cancer patients aged 0 to 23 years, identifying congenital anomalies before the onset of the tumor in 141 (13% of cases) out of a sample of 1107 patients; specifically, the following pathologies were observed: movement disorders, obstructive hydrocephalus, Arnold–Chiari malformation, spina bifida, cerebral palsy, epilepsy and, less frequently, cardiovascular, gastrointestinal or genitourinary tract anomalies. Congenital anomalies of the CNS were found to be associated with CNS tumors (less frequently with astrocytomas and ependymomas, more frequently with gliomas and neuroblastomas), especially in males aged less than 5 years [43].

A correlation between neurofibromatosis, tumors and neurodevelopmental disorders is also known; in particular, a compromise in the formation process of dendritic spines has been observed [44].

Genetic predisposition, therefore, plays a key role in this area, and should also be taken into consideration for chemotherapy treatment in these children. Indeed, recent studies have highlighted specific genetic polymorphisms connected to different alterations of cognitive performances or behavioral disorders; a role for genes related to oxidative stress and neuro-inflammation is believed to contribute to the neurocognitive decline associated with chemotherapy in children with ALL [31,45,46]. It is possible that some DNA polymorphisms related to brain development, APOE and brain-derived neurotrophic factor (BDNF) may provide protection against neurotoxicity during development; however, further studies are needed because it is not clear which genotypes and immunological mechanisms are involved in chemotherapy-induced neurotoxicity [9]. Finally, radiation therapy is suspected to induce a shortening of telomeres [47].

## 2. The Role of Sleep Disorders in Children with Cancer and Neurodevelopmental Disorders

The close correlation between sleep disturbances and pediatric cancer [2,11,12] and how the treatment of tumors can negatively impact children’s health, even after recovery from cancer, has recently been highlighted [3,7,9,10]; however, papers on this topic are not homogeneous and evidence is scattered around in several observational studies and case series and reports that are briefly described below.

Since pediatric brain tumor survivors are at risk of both sleep disturbance and neurocognitive impairment, improving sleep may be a way to promote neurocognitive functioning in these children, for whom adequate interventions are currently scarce [48]. It is also true that a knowledge of sleep disorders in children with neurodevelopmental pathologies predisposed to develop cancer can improve their health and quality of life; however, there are only few studies in the literature that have evaluated this aspect, highlighting a high prevalence of sleep disturbances in tuberous sclerosis (74% of cases) and in autism spectrum disorder (50–80% of cases) [49].

One of the first attempts to demonstrate that neurocognitive function (attention, memory and processing speed deficits) in long-term survivors of childhood cancer appears to be particularly vulnerable to the effects of fatigue and sleep fragmentation dates back to 2011, and suggested that sleep hygiene could be a valid tool to improve neurocognitive outcome [14].

As previously said, neoplasms in children are associated with neurodevelopmental problems related to neuroinflammation [36]; there is growing evidence that systemic inflammation is associated with poor sleep quality [50]; and sleep duration is linked to serum concentrations of C-reactive protein (CRP), tumor necrosis factor-alpha (TNF-α) and interleukin [51]. Table 2 summarizes some significant aspects of the importance of sleep for the developing brain.

### 2.1. Inflammation and Oxidative Stress

Only one study evaluated the association of inflammation and oxidative stress biomarkers with neurocognitive outcomes and sleep quality in long-term survival children with ALL [13]. Females with high levels of CRP, interleukin-6 and interleukin-1β (IL-1β) showed greater neurocognitive and behavioral involvement, while higher levels of IL-1β were associated with cognitive impairment and longer sleep duration; females seemed to be more affected than males in both cognitive and sleep disturbances. Moreover, a longer sleep duration was associated with a worsening of the executive functions of the frontal lobes in females but not in males [13]. Symptoms of fatigue and sleep problems might be aggravated by the impact of immune activation and the interruption of circadian rhythms secondary to the adrenal insufficiency subsequent to the treatment of cancer; however, further data are needed, also due to the limited number of cases studied [13].

There is growing evidence supporting that mediators of inflammation and immune cells are the main regulators of the sexual differentiation of the brain, in addition to neurotransmitters and sex hormones [61]; on the other hand, the importance of good sleep quality for improving the functioning of the immune system is increasingly evident [56].

Sleep deprivation causes an increase in inflammatory cytokine levels, especially interleukin 6 (IL-6) and tumor necrosis factor alpha (TNF-α) a reduction in the number of T-helper lymphocytes (CD 3+ and CD 4+), T-cytotoxic lymphocytes (CD 8+) and natural killer cells (NK) has been observed in insomniacs; under physiological conditions, sleep induction and the first stages after falling asleep are related to an increase in IL-1 levels: both the latter and TNF-α decrease during the subsequent sleep stages, while other cytokines (IL-2, IL-6, IL-8, IL-15 and IL-18) promote non-rapid-eye-movement (NREM) sleep and regulate body temperature. It can therefore be deduced how important good sleep quality is for the integrity of the immune system; in addition, the communication network between the immune system and the neuroendocrine system allows the body to maintain homeostasis, especially when it must respond to an external stimulus, such as an infection [56].

Most sleep disturbances in cancer patients are associated with an activation of the inflammatory state, even during chemotherapy: cytokines activate microglia (through the humoral and/or neural pathway), which can in turn induce an astrocyte neurotoxic reaction. Tumors produce IL 1-β in high quantities, which inhibits rapid-eye-movement (REM) and promotes NREM sleep and influences numerous neurotransmitters involved in sleep regulation (adenosine, prostaglandins, nitric oxide, GABA). IL-6 also seems to reduce REM sleep and increase slow-wave sleep; the same role seems to be played by TNF-α [57]. Hormones are also involved in the close connection between sleep and tumors: ghrelin, related to an increase in tumor progression and a reduction in survival, could act on orexinergic neurons by activating them; leptin, involved in the proliferation of cancer cells, can induce the production of IL-6 and TNF-α; moreover, it seems to activate hypothalamic neurons, in turn connected with orexinergic neurons. Finally, neurons expressing calcitonin-gene-related peptide are sensitive to changes in pCO_2_ and are involved in the onset of arousals and awakening [57].

The interaction between sleep and tumors is also of fundamental importance for therapeutic purposes, as the response to chemotherapy and immunotherapy requires adequate functioning of the immune system which is influenced by sleep. It is known how the immune response undergoes maturation in the course of neonatal, infant and adult life and how important it is to preserve its development and functioning in order to avoid the risk of infections, immunological disorders and cancer [58]; immune activation, inflammation and various other conditions that cause unwanted microglial activity might seriously impair learning, memory and other essential cognitive functions [52]. Therefore, it is essential to preserve the functioning of the immune system in children with cancer with an adequate management of sleep disorders.

### 2.2. Neurogenesis and Synaptic Plasticity

A major challenge in counteracting the consequences of childhood cancer on neurological development is, therefore, the prevention of cancer-induced neuronal damage and its treatment. There is a complex interaction between neurogenesis of the neurons within the dentate gyrus of the hippocampus, memory function, neuronal plasticity and sleep; as an example, attention to sleep characteristics is indicated for the treatment of cognitive decline [53].

Several studies have shown that sleep improves learning and memory, enhancing synaptic plasticity at the hippocampus level, where among other things there are stem cells (in correspondence with the dentate gyrus); memory traces are consolidated here during the slow-wave sleep stages. Therefore, it is important to ensure good sleep quality and adequate duration of deep sleep, so that this process and sleep homeostasis are guaranteed. In fact, it has been shown that sleep fragmentation and deprivation and circadian rhythm alterations are also associated with neurodegenerative processes, as well as tumor pathologies, through an increase in oxidative stress and inflammatory mechanisms, which can trigger cell degeneration cascade processes [59].

As previously described, sometimes it is chemotherapy itself that induces neuronal damage, which is even more serious if it happens in children who are affected by neurodevelopmental disorders as well as by neoplastic pathologies. Among other things, it seems that the brain area most damaged by cancer and by chemotherapy treatment in children is precisely the hippocampus [7,41]. Thus, it may be essential to increase sleep quality as an effective therapy that can counteract neuronal degeneration processes.

### 2.3. The Role of Melatonin

The treatment of sleep disturbances can also ensure adequate production of endogenous melatonin, a hormone with anti-tumor and anti-inflammatory functions, which stimulates the proliferation of stem cells [15,54,55]; the regularization of circadian rhythms is important for a correct functioning of the hypothalamic–pituitary–adrenal axis, unfortunately often involved in pediatric neoplasms [37,41,42], with consequent improvement of the production of hormones synthesized by these metabolic pathways, also important for the processes of growth of the child.

### 2.4. Interaction between Sleep and Tau Protein

Regarding severe cognitive decline, for its prevention, it is important to underline that sleep deprivation can induce an increase in tau protein, the accumulation of which can cause neurodegeneration [62]. As previously mentioned, in children with ALL undergoing lumbar puncture, an increase in the levels of tau protein (which is one of the markers of neurodegeneration) has been demonstrated both before and after the start of chemotherapy treatment, thus being able to indicate a possible tumor-induced (or treatment-induced) neurodegenerative process [36]. Thus, if sleep deprivation is also related to an increase in tau protein, a failure to treat sleep disturbances in children with leukemia can cause further worsening of neurodevelopment. This aspect is even more serious if we also consider that the accumulation of tau protein is a process that is perpetuated over time and is in fact linked in adults to neurodegenerative processes that occur in some types of dementia, such as Alzheimer’s disease and other tauopathies [63].

### 2.5. Brain Connectivity

Furthermore, the maturation of slow waves in childhood seems to be a new marker for monitoring brain connections, giving to sleep the role of a major player in brain maturation processes [60]. Myelination processes that occur from childhood to adolescence, leading to the maturation of nerve fibers and allowing neurodevelopmental mechanisms, take place especially during sleep, which, therefore, plays a key role in brain maturation and learning; in the course of pediatric cancer, a widespread involvement is observed not only of the gray matter, but also of the white matter [3,10,30,36], with the consequent disruption of brain connectivity, impaired learning processes and reduced cognitive performance [34,35]. Good sleep quality, with an adequate representation of slow-wave sleep, is therefore important for brain connectivity and neurodevelopment.

As a consequence of all the above considerations, the relationship and interactions between pediatric cancer, chemotherapy, sleep and CNS development/damage and cognitive function is complex and schematically represented in Figure 1.

## 3. The Effect of Sleep Disorder Treatment on Neurodevelopmental Disorders in Children with Cancer

Studies on the treatment of sleep disorders in children with oncological problems and neurodevelopmental disorders are very few and concern the usefulness of CBT and melatonin; in the latter case, the greatest evidence is provided by studies conducted on adults [15,16,53,54,60,62,63,64]. Further studies in this regard could be very useful for improving the therapeutic outcome and quality of life in children with cancer and cognitive problems.

### 3.1. Cognitive Behavioral Therapy

The importance of CBT for sleep disturbances in children with malignancies has also been highlighted; however, standardized protocols are lacking and only a limited number of studies are available [64].

A very recent study has evaluated the effects of the treatment of sleep disturbances in children with CNS tumors exposed to high doses of chemotherapy, hypothesizing that the treatment of sleep disturbances and the protection of endogenous melatonin production might be followed by a better outcome of autologous stem cell transplantation too [15]. The same investigation suggested the usefulness of chronopharmacology with chemotherapy agents, as also reported by other studies [65]; this chrono-chemotherapy approach is innovative and exploits the circadian biology of individual tumors and the specific circadian variations influencing the pharmacokinetics and pharmacodynamics of drugs, and consequently proving effective in reducing the doses of some drugs with the same efficacy, and, finally, ensuring a better toxicity profile. However, these are pioneering studies conducted in adults and not yet attempted in the pediatric population.

Another work highlighted that sleep disturbances arise in half of children with neoplasms and neurocognitive problems, with worsening of executive functions, and underlined the importance of the inclusion of CBT of sleep disturbances in the treatment protocols of children with neoplastic problems [16]. The importance of CBT in these children was also demonstrated in another recent study, which assessed sleep disturbances such as insomnia, obstructive sleep apnea syndrome, excessive daytime sleepiness and circadian rhythm disturbances in children with tumors; a positive outcome on psychological disorders was also reported [66].

CBT represents the first approach in different types of sleep disorders, first of all insomnia, according to international guidelines, even before any drug treatment. Children suffering from oncological problems are fragile and the concomitant presence of cognitive problems and mood disorders should be considered. Often, because of the impossibility of using a pharmacological support (given the age of the patients and the associated clinical conditions), it is of fundamental importance to assess the possible presence of different sleep disorders and to use CBT before chemotherapy, during it and during the follow-up period, to ensure adequate and timely management of sleep quality, improve health and adherence to chemotherapy, and support cognitive development. It would be useful to create ad-hoc protocols for children with cancer, especially with neurodevelopmental problems.

### 3.2. Melatonin

Melatonin is a promising treatment for sleep disorders in children (important for regulating the sleep–wake cycle); melatonin seems to be a powerful free radical scavenger and has an anti-tumor action [54,55,67]. Melatonin stimulates the apoptosis of cancer cells and their anti-growth signal, enhances the immune system, and inhibits several active mechanisms in carcinogenesis, including genomic instability, angiogenesis, the development of metastasis (limiting the entrance of neoplastic cells in the vascular system), and the inflammatory processes promoted by tumors [54].

Melatonin is a powerful free radical scavenger and has a chelating action, and therefore reduces oxidative stress and inflammatory reactions and stabilizes cell membranes. It has been hypothesized that melatonin can improve the survival of stem cells and facilitate their differentiation [55]; it was also shown that preserving endogenous melatonin production improves response to autologous stem cell transplantation [15].

Melatonin seems to improve the response to chemotherapy treatment and reduce drug resistance to anti-tumor drugs. It also seems to play a role in the reduction of the acute and long-term toxicity of chemotherapy, thus suggesting the importance of its use in association with chemotherapy [67]. Current research on melatonin supplementation during cancer treatment is evaluating its possible role in improving sleep, appetite, the effectiveness of radiation therapy and immunological and inflammatory markers, as well as a reduction in neurocognitive deficits [59,68].

In a meta-analysis, which evaluated 21 trials conducted on cancer patients, the effects of adding melatonin to chemotherapy treatment were evaluated, revealing that patients who had also taken melatonin had a better therapeutic response to cancer remission and an improved one-year survival [59]. In this case, studies have also only been carried out in adults.

Melatonin is indicated for the treatment of insomnia and circadian rhythm disorders with delayed sleep phase. It is very effective if administered 3–5 h before the physiological dim light melatonin onset. Many children with neurodevelopmental disorders (such as autism spectrum disorders, attention deficit/hyperactivity disorder and intellectual disabilities) can benefit from melatonin treatment, which leads to a reduction in sleep latency and increases total sleep time. No serious adverse reactions have been identified following the intake of melatonin [69].

### 3.3. Other Treatment Options

Sleep disorders are very common in children with neurodevelopmental disorders; however, there are no drugs approved for the treatment of children’s sleep disorders by the United States Food and Drug Administration. Therefore, many pharmaceuticals are prescribed off-label [70]. Treatment in children must be personalized and consider multiple factors, such as the type and severity of sleep disorder and the comorbidities [70], especially in children with oncological pathology, considering the side effects of anti-tumor drugs and possible interactions with these. In a recent review, which assessed the various therapeutic options for the pharmacological treatment of sleep disorders in children with neurodevelopmental disorders, it emerged that the use of certain drugs such as gabapentin, clonidine, trazodone and mirtazapine could be therapeutic options and that, supplementation with iron, vitamin D and 5-hydroxy tryptophan could be useful; however, further studies are needed to evaluate doses and tolerability [70]. Regarding iron supplementation, a good response to oral iron intake has been demonstrated for the treatment of restless legs syndrome in children [71], especially in long-term treatment [72], and this data assumes a particular relevance if we consider the prevalence of this disorder in some neurodevelopmental disorders, such as attention-deficit hyperactivity disorder [73], and the high frequency with which problems of anemia occur in hematological malignancies.

### 3.4. Ventilatory Treatment of Respiratory Disorders in Sleep

Obstructive sleep apnea (OSA) is very common in children [74] and can be associated with neurocognitive problems or neurodevelopmental disorders, such as attention deficit/hyperactivity disorder and learning problems [75,76]. OSA determines intermittent hypoxia, endothelial dysfunction, systemic inflammation and, therefore, cell damage and is related to cardiovascular problems, diabetes, high blood pressure and metabolic syndrome. In addition, the excessive daytime sleepiness caused by this disease can negatively impact on the child’s learning, behavior and school performance [75] and cause an alteration of the cerebral gray matter in the areas involved in learning and emotional functions [77]. Thus it is important to treat this pathology, often also found in children with oncological pathologies [12,78], in whom neurocognitive problems can arise both as a consequence of cancer or of its treatment, as previously described [17,18,31,32,34,35]. As mentioned above, both OSA and cancer can cause structural changes in the brain, especially in the earliest stages of the child’s development [3,10,29,30,31,32,77]. It has been shown that OSA ventilatory treatment, as well as surgical treatment (through adenotonsillectomy), can induce an improvement in attention, social interaction, behavior and cognitive skills [79]. However, good adherence by the patient is necessary for the ventilatory treatment in order to be effective, which can be difficult, especially with children with neurocognitive problems. It is therefore necessary to optimize strategies together with the caregiver to ensure good compliance of the child with ventilatory treatment [80].

## 4. Conclusions

Considering the improvement in the last few years in terms of the survival of pediatric malignancies and the high prevalence of cognitive problems in these children, due to both the effects of pediatric tumors themselves and their treatment, it is clear that in-depth knowledge and correct management of sleep disorders may help improve the health and quality of life of children with cancer and their families. New, well-planned and controlled trials are now warranted for the design of targeted therapeutic approaches and standardized protocols.

## Figures and Tables

**Table 1:** Biological damage of the central nervous system observed in pediatric cancer.

- Damage of the hippocampus and other areas involved in memory emotion circuits (amygdala, thalamus, striatum, substantia nigra/ventral tegmental area) [10,29,30,31,32] - Dendrite and axonal growth damage [10] damage of prefrontal functions [29,31,32,35,36] brain connectivity disruption [34] leukoencephalopathy [13,36] - Increase in tau protein (axonal damage and neurodegeneration), increase in GFAP (associated with attention deficit) [36] neuroinflammation (increase in C-reactive protein, IL, and tumor necrosis factor-α) [9,26,31,32] hypothalamic–pituitary–adrenal axis alteration [37]

**Table 2:** The identified roles of sleep in the development of the central nervous system and cancer.

- Stimulation of neurogenesis in the hippocampal dentate gyrus [15,52,53,54,55] - Improvement of neuronal plasticity [52,53] - Immunity system potentiation [13] - Sleep slow-wave maturation stimulates brain connectivity [51,56,57,58,59] - Sleep deprivation causes an increase in tau protein [60] - Melatonin sleep/wake cycle regulation [37,42,59] anti-tumor action [15,54,55] powerful free radical scavenger [15,54,55]

## References

[1] Howlader N., Noone A.M., Krapcho M., Miller D., Bishop K., Altekruse S.F., Kosary C.L., Yu M., Ruhl J., Tatalovich Z. (2016). SEER Cancer Statistics Review, 1975–2013.

[2] Walter L.M., Nixon G.M., Davey M.J., Downie P.A., Horne R.S. (2015). Sleep and fatigue in pediatric oncology: A review of the literature. Sleep Med. Rev..

[3] Cheung Y.T., Krull K.R. (2015). Neurocognitive outcomes in long-term survivors of childhood acute lymphoblastic leukemia treated on contemporary treatment protocols: A systematic review. Neurosci. Biobehav. Rev..

[4] Cheung Y.T., Sabin N.D., Reddick W.E., Bhojwani D., Liu W., Brinkman T.M., Glass J.O., Hwang S.N., Srivastava D., Pui C.H. (2016). Leukoencephalopathy and long-term neurobehavioural, neurocognitive, and brain imaging outcomes in survivors of childhood acute lymphoblastic leukaemia treated with chemotherapy: A longitudinal analysis. Lancet Haematol..

[5] Butler R.W., Haser J.K. (2006). Neurocognitive effects of treatment for childhood cancer. Ment. Retard. Dev. Disabil. Res. Rev..

[6] Ashford J., Schoffstall C., Reddick W.E., Leone C., Laningham F.H., Glass J.O., Pei D., Cheng C., Pui C.H., Conklin H.M. (2010). Attention and working memory abilities in children treated for acute lymphoblastic leukemia. Cancer.

[7] Marusak H.A., Iadipaolo A.S., Harper F.W., Elrahal F., Taub J.W., Goldberg E., Rabinak C.A. (2018). Neurodevelopmental consequences of pediatric cancer and its treatment: Applying an early adversity framework to understanding cognitive, behavioral, and emotional outcomes. Neuropsychol. Rev..

[8] Duffner P.K. (2010). Risk factors for cognitive decline in children treated for brain tumors. Eur. J. Paediatr. Neurol. Off. J. Eur. Paediatr. Neurol. Soc..

[9] Sleurs C., Deprez S., Emsell L., Lemiere J., Uyttebroeck A. (2016). Chemotherapy-induced neurotoxicity in pediatric solid non-CNS tumor patients: An update on current state of research and recommended future directions. Crit. Rev. Oncol. Hematol..

[10] de Ruiter M.A., van Mourik R., Schouten-van Meeteren A.Y., Grootenhuis M.A., Oosterlaan J. (2013). Neurocognitive consequences of a paediatric brain tumour and its treatment: A meta-analysis. Dev. Med. Child Neurol..

[11] Kaleyias J., Manley P., Kothare S.V. (2012). Sleep disorders in children with cancer. Semin. Pediatric Neurol..

[12] Olson K. (2014). Sleep-related disturbances among adolescents with cancer: A systematic review. Sleep Med..

[13] Cheung Y.T., Brinkman T.M., Mulrooney D.A., Mzayek Y., Liu W., Banerjee P., Panoskaltsis-Mortari A., Srivastava D., Pui C.H., Robison L.L. (2017). Impact of sleep, fatigue, and systemic inflammation on neurocognitive and behavioral outcomes in long-term survivors of childhood acute lymphoblastic leukemia. Cancer.

[14] Clanton N.R., Klosky J.L., Li C., Jain N., Srivastava D.K., Mulrooney D., Zeltzer L., Stovall M., Robison L.L., Krull K.R. (2011). Fatigue, vitality, sleep, and neurocognitive functioning in adult survivors of childhood cancer: A report from the Childhood Cancer Survivor Study. Cancer.

[15] Rogers V.E., Zhu S., Ancoli-Israel S., Liu L., Mandrell B.N., Hinds P.S. (2019). A pilot randomized controlled trial to improve sleep and fatigue in children with central nervous system tumors hospitalized for high-dose chemotherapy. Pediatric Blood Cancer.

[16] van Kooten J., Maurice-Stam H., Schouten A.Y.N., van Vuurden D.G., Granzen B., Gidding C., de Ruiter M.A., van Litsenburg R.R.L., Grootenhuis M.A. (2019). High occurrence of sleep problems in survivors of a childhood brain tumor with neurocognitive complaints: The association with psychosocial and behavioral executive functioning. Pediatric Blood Cancer.

[17] Conklin H.M., Krull K.R., Reddick W.E., Pei D., Cheng C., Pui C.H. (2012). Cognitive outcomes following contemporary treatment without cranial irradiation for childhood acute lymphoblastic leukemia. J. Natl. Cancer Inst..

[18] Castellino S.M., Ullrich N.J., Whelen M.J., Lange B.J. (2014). Developing interventions for cancer-related cognitive dysfunction in childhood cancer survivors. J. Natl. Cancer Inst..

[19] Price J., Kassam-Adams N., Alderfer M.A., Christofferson J., Kazak A.E. (2016). Systematic Review: A Reevaluation and Update of the Integrative (Trajectory) Model of Pediatric Medical Traumatic Stress. J. Pediatric Psychol..

[20] Turner J.K., Hutchinson A., Wilson C. (2018). Correlates of post-traumatic growth following childhood and adolescent cancer: A systematic review and meta-analysis. Psycho-oncology.

[21] Dutil C., Walsh J.J., Featherstone R.B., Gunnell K.E., Tremblay M.S., Gruber R., Weiss S.K., Cote K.A., Sampson M., Chaput J.P. (2018). Influence of sleep on developing brain functions and structures in children and adolescents: A systematic review. Sleep Med. Rev..

[22] Robinson J.L., Erath S.A., Kana R.K., El-Sheikh M. (2018). Neurophysiological differences in the adolescent brain following a single night of restricted sleep—A 7T fMRI study. Dev. Cogn. Neurosci..

[23] Qiu D., Leung L.H., Kwong D.L., Chan G.C., Khong P.L. (2006). Mapping radiation dose distribution on the fractional anisotropy map: Applications in the assessment of treatment-induced white matter injury. NeuroImage.

[24] Reddick W.E., Glass J.O., Helton K.J., Langston J.W., Li C.S., Pui C.H. (2005). A quantitative MR imaging assessment of leukoencephalopathy in children treated for acute lymphoblastic leukemia without irradiation. Am. J. Neuroradiol..

[25] Reddick W.E., Glass J.O., Helton K.J., Langston J.W., Xiong X., Wu S., Pui C.H. (2005). Prevalence of leukoencephalopathy in children treated for acute lymphoblastic leukemia with high-dose methotrexate. Am. J. Neuroradiol..

[26] Bornstein M.H., Scrimin S., Putnick D.L., Capello F., Haynes O.M., de Falco S., Carli M., Pillon M. (2012). Neurodevelopmental functioning in very young children undergoing treatment for non-CNS cancers. J. Pediatric Psychol..

[27] Patel S.K., Mullins W.A., O’Neil S.H., Wilson K. (2011). Neuropsychological differences between survivors of supratentorial and infratentorial brain tumours. J. Intellect. Disabil. Res..

[28] Ris M.D., Packer R., Goldwein J., Jones-Wallace D., Boyett J.M. (2001). Intellectual outcome after reduced-dose radiation therapy plus adjuvant chemotherapy for medulloblastoma: A Children’s Cancer Group study. J. Clin. Oncol. Off. J. Am. Soc. Clin. Oncol..

[29] Riem M.M., Alink L.R., Out D., Van Ijzendoorn M.H., Bakermans-Kranenburg M.J. (2015). Beating the brain about abuse: Empirical and meta-analytic studies of the association between maltreatment and hippocampal volume across childhood and adolescence. Dev. Psychopathol..

[30] Monje M., Dietrich J. (2012). Cognitive side effects of cancer therapy demonstrate a functional role for adult neurogenesis. Behav. Brain Res..

[31] Cole P.D., Finkelstein Y., Stevenson K.E., Blonquist T.M., Vijayanathan V., Silverman L.B., Neuberg D.S., Sallan S.E., Robaey P., Waber D.P. (2015). Polymorphisms in Genes Related to Oxidative Stress Are Associated With Inferior Cognitive Function After Therapy for Childhood Acute Lymphoblastic Leukemia. J. Clin. Oncol. Off. J. Am. Soc. Clin. Oncol..

[32] Dietrich J., Prust M., Kaiser J. (2015). Chemotherapy, cognitive impairment and hippocampal toxicity. Neuroscience.

[33] Krull K.R., Hardy K.K., Kahalley L.S., Schuitema I., Kesler S.R. (2018). Neurocognitive Outcomes and Interventions in Long-Term Survivors of Childhood Cancer. J. Clin. Oncol. Off. J. Am. Soc. Clin. Oncol..

[34] Kesler S.R., Ogg R., Reddick W.E., Phillips N., Scoggins M., Glass J.O., Cheung Y.T., Pui C.H., Robison L.L., Hudson M.M. (2018). Brain Network Connectivity and Executive Function in Long-Term Survivors of Childhood Acute Lymphoblastic Leukemia. Brain Connect..

[35] Fellah S., Cheung Y.T., Scoggins M.A., Zou P., Sabin N.D., Pui C.H., Robison L.L., Hudson M.M., Ogg R.J., Krull K.R. (2019). Brain Activity Associated With Attention Deficits Following Chemotherapy for Childhood Acute Lymphoblastic Leukemia. J. Natl. Cancer Inst..

[36] Cheung Y.T., Khan R.B., Liu W., Brinkman T.M., Edelmann M.N., Reddick W.E., Pei D., Panoskaltsis-Mortari A., Srivastava D., Cheng C. (2018). Association of Cerebrospinal Fluid Biomarkers of Central Nervous System Injury With Neurocognitive and Brain Imaging Outcomes in Children Receiving Chemotherapy for Acute Lymphoblastic Leukemia. JAMA Oncol..

[37] Gapstur R., Gross C.R., Ness K. (2009). Factors associated with sleep-wake disturbances in child and adult survivors of pediatric brain tumors: A review. Oncol. Nurs. Forum.

[38] Kesler S.R., Lacayo N.J., Jo B. (2011). A pilot study of an online cognitive rehabilitation program for executive function skills in children with cancer-related brain injury. Brain Inj..

[39] Riggs L., Piscione J., Laughlin S., Cunningham T., Timmons B.W., Courneya K.S., Bartels U., Skocic J., de Medeiros C., Liu F. (2017). Exercise training for neural recovery in a restricted sample of pediatric brain tumor survivors: A controlled clinical trial with crossover of training versus no training. Neuro-oncology.

[40] Baum K.T., Powell S.K., Jacobson L.A., Gragert M.N., Janzen L.A., Paltin I., Rey-Casserly C.M., Wilkening G.N. (2017). Implementing guidelines: Proposed definitions of neuropsychology services in pediatric oncology. Pediatric Blood Cancer.

[41] Kohman R.A., Rhodes J.S. (2013). Neurogenesis, inflammation and behavior. Brain Behav. Immun..

[42] Zhang J., Walsh M.F., Wu G., Edmonson M.N., Gruber T.A., Easton J., Hedges D., Ma X., Zhou X., Yergeau D.A. (2015). Germline Mutations in Predisposition Genes in Pediatric Cancer. N. Engl. J. Med..

[43] Wong-Siegel J.R., Johnson K.J., Gettinger K., Cousins N., McAmis N., Zamarione A., Druley T.E. (2017). Congenital neurodevelopmental anomalies in pediatric and young adult cancer. Am. J. Med Genet. Part A.

[44] Hsueh Y.P. (2012). From neurodevelopment to neurodegeneration: The interaction of neurofibromin and valosin-containing protein/p97 in regulation of dendritic spine formation. J. Biomed. Sci..

[45] Krull K.R., Bhojwani D., Conklin H.M., Pei D., Cheng C., Reddick W.E., Sandlund J.T., Pui C.H. (2013). Genetic mediators of neurocognitive outcomes in survivors of childhood acute lymphoblastic leukemia. J. Clin. Oncol. Off. J. Am. Soc. Clin. Oncol..

[46] Krull K.R., Hockenberry M.J., Miketova P., Carey M., Moore I.M. (2013). Chemotherapy-related changes in central nervous system phospholipids and neurocognitive function in childhood acute lymphoblastic leukemia. Leuk. Lymphoma.

[47] Shim G., Ricoul M., Hempel W.M., Azzam E.I., Sabatier L. (2014). Crosstalk between telomere maintenance and radiation effects: A key player in the process of radiation-induced carcinogenesis. Mutat. Res. Rev. Mutat. Res..

[48] de Ruiter M.A., Oosterlaan J., Schouten-van Meeteren A.Y., Maurice-Stam H., van Vuurden D.G., Gidding C., Beek L.R., Granzen B., Caron H.N., Grootenhuis M.A. (2016). Neurofeedback ineffective in paediatric brain tumour survivors: Results of a double-blind randomised placebo-controlled trial. Eur. J. Cancer.

[49] Trickett J., Heald M., Oliver C., Richards C. (2018). A cross-syndrome cohort comparison of sleep disturbance in children with Smith-Magenis syndrome, Angelman syndrome, autism spectrum disorder and tuberous sclerosis complex. J. Neurodev. Disord..

[50] Schubert C., Hong S., Natarajan L., Mills P.J., Dimsdale J.E. (2007). The association between fatigue and inflammatory marker levels in cancer patients: A quantitative review. Brain Behav. Immun..

[51] Kesler S., Janelsins M., Koovakkattu D., Palesh O., Mustian K., Morrow G., Dhabhar F.S. (2013). Reduced hippocampal volume and verbal memory performance associated with interleukin-6 and tumor necrosis factor-alpha levels in chemotherapy-treated breast cancer survivors. Brain Behav. Immun..

[52] Tay T.L., Savage J.C., Hui C.W., Bisht K., Tremblay M.E. (2017). Microglia across the lifespan: From origin to function in brain development, plasticity and cognition. J. Physiol..

[53] Koyanagi I., Akers K.G., Vergara P., Srinivasan S., Sakurai T., Sakaguchi M. (2019). Memory consolidation during sleep and adult hippocampal neurogenesis. Neural Regen. Res..

[54] Talib W.H. (2018). Melatonin and Cancer Hallmarks. Molecules.

[55] Lee M.S., Yin T.C., Sung P.H., Chiang J.Y., Sun C.K., Yip H.K. (2017). Melatonin enhances survival and preserves functional integrity of stem cells: A review. J. Pineal Res..

[56] Asif N., Iqbal R., Nazir C.F. (2017). Human immune system during sleep. Am. J. Clin. Exp. Immunol..

[57] Walker W.H., Borniger J.C. (2019). Molecular Mechanisms of Cancer-Induced Sleep Disruption. Int. J. Mol. Sci..

[58] Simon A.K., Hollander G.A., McMichael A. (2015). Evolution of the immune system in humans from infancy to old age. Proc. R. Soc. B Biol. Sci..

[59] Seely D., Wu P., Fritz H., Kennedy D.A., Tsui T., Seely A.J., Mills E. (2012). Melatonin as adjuvant cancer care with and without chemotherapy: A systematic review and meta-analysis of randomized trials. Integr. Cancer Ther..

[60] Kurth S., Riedner B.A., Dean D.C., O’Muircheartaigh J., Huber R., Jenni O.G., Deoni S.C.L., LeBourgeois M.K. (2017). Traveling Slow Oscillations During Sleep: A Marker of Brain Connectivity in Childhood. Sleep.

[61] McCarthy M.M., Pickett L.A., VanRyzin J.W., Kight K.E. (2015). Surprising origins of sex differences in the brain. Horm. Behav..

[62] Holth J.K., Fritschi S.K., Wang C., Pedersen N.P., Cirrito J.R., Mahan T.E., Finn M.B., Manis M., Geerling J.C., Fuller P.M. (2019). The sleep-wake cycle regulates brain interstitial fluid tau in mice and CSF tau in humans. Science.

[63] Scheltens P., Blennow K., Breteler M.M., de Strooper B., Frisoni G.B., Salloway S., Van der Flier W.M. (2016). Alzheimer’s disease. Lancet.

[64] Merz E.L., Tomfohr-Madsen L. (2018). Sleep Disruption in Pediatric Cancer Survivors: Conceptual Framework and Opportunities for Clinical Assessment and Behavioral Treatment. Am. J. Lifestyle Med..

[65] Ballesta A., Innominato P.F., Dallmann R., Rand D.A., Levi F.A. (2017). Systems Chronotherapeutics. Pharmacol. Rev..

[66] Daniel L.C., Wang M., Mulrooney D.A., Srivastava D.K., Schwartz L.A., Edelstein K., Brinkman T.M., Zhou E.S., Howell R.M., Gibson T.M. (2019). Sleep, emotional distress, and physical health in survivors of childhood cancer: A report from the Childhood Cancer Survivor Study. Psycho-oncology.

[67] Reiter R.J., Rosales-Corral S.A., Tan D.X., Acuna-Castroviejo D., Qin L., Yang S.F., Xu K. (2017). Melatonin, a Full Service Anti-Cancer Agent: Inhibition of Initiation, Progression and Metastasis. Int. J. Mol. Sci..

[68] Wang Y.M., Jin B.Z., Ai F., Duan C.H., Lu Y.Z., Dong T.F., Fu Q.L. (2012). The efficacy and safety of melatonin in concurrent chemotherapy or radiotherapy for solid tumors: A meta-analysis of randomized controlled trials. Cancer Chemother. Pharmacol..

[69] Bruni O., Alonso-Alconada D., Besag F., Biran V., Braam W., Cortese S., Moavero R., Parisi P., Smits M., Van der Heijden K. (2015). Current role of melatonin in pediatric neurology: Clinical recommendations. Eur. J. Paediatr. Neurol. Off. J. Eur. Paediatr. Neurol. Soc..

[70] Bruni O., Angriman M., Melegari M.G., Ferri R. (2019). Pharmacotherapeutic management of sleep disorders in children with neurodevelopmental disorders. Expert Opin. Pharmacother..

[71] DelRosso L., Bruni O. (2019). Treatment of pediatric restless legs syndrome. Adv. Pharmacol..

[72] DelRosso L.M., Yi T., Chan J.H.M., Wrede J.E., Lockhart C.T., Ferri R. (2020). Determinants of ferritin response to oral iron supplementation in children with sleep movement disorders. Sleep.

[73] Angriman M., Cortese S., Bruni O. (2017). Somatic and neuropsychiatric comorbidities in pediatric restless legs syndrome: A systematic review of the literature. Sleep Med. Rev..

[74] Lumeng J.C., Chervin R.D. (2008). Epidemiology of pediatric obstructive sleep apnea. Proc. Am. Thorac. Soc..

[75] Lal C., Strange C., Bachman D. (2012). Neurocognitive impairment in obstructive sleep apnea. Chest.

[76] Hvolby A. (2015). Associations of sleep disturbance with ADHD: Implications for treatment. Atten. Deficit Hyperact. Disord..

[77] Philby M.F., Macey P.M., Ma R.A., Kumar R., Gozal D., Kheirandish-Gozal L. (2017). Reduced Regional Grey Matter Volumes in Pediatric Obstructive Sleep Apnea. Sci. Rep..

[78] Russo S., Fardell J.E., Signorelli C., Wakefield C.E., McLoone J.K., Cohn R.J. (2016). Sleep Disturbances in Childhood Cancer Survivors. Pediatric Blood Cancer.

[79] Konstantinopoulou S., Tapia I.E. (2016). Neurocognitive and Behavioural Outcomes Following Intervention for Obstructive Sleep Apnoea Syndrome in Children. Paediatr. Respir. Rev..

[80] Xanthopoulos M.S., Kim J.Y., Blechner M., Chang M.Y., Menello M.K., Brown C., Matthews E., Weaver T.E., Shults J., Marcus C.L. (2017). Self-Efficacy and Short-Term Adherence to Continuous Positive Airway Pressure Treatment in Children. Sleep.

